# Risk Factors for Acute Pancreatitis Following Intragastric Balloon Insertion: A 7-Year Retrospective Cohort Study

**DOI:** 10.1007/s11695-024-07647-x

**Published:** 2025-01-14

**Authors:** Yousef Yahia, Joud Abuodeh, Prem Chandra, Ethar Mohamed, Anas Zayad, Leen AbuAfifeh

**Affiliations:** Hamad Medical Corporation, Doha, Qatar

**Keywords:** Intragastric balloon, Acute pancreatitis, Obesity, Weight loss, Risk factors, Complications, Orbera balloon

## Abstract

**Background:**

Acute pancreatitis (AP) is a rare but serious complication of intragastric balloon (IGB) therapy. Despite the popularity of IGBs for weight loss, the incidence and risk factors of AP post-IGB insertion are not well understood. This study aimed to identify potential predictors and risk factors of AP in IGB patients.

**Methods:**

A retrospective time-to-event study was conducted over 7 years, encompassing patients who received IGBs between January 2017 and 2024. Cox regression analyses were performed to identify risk factors. The incidence of AP was evaluated as a secondary outcome. Patients were categorized into the AP and non-AP groups at a 1:3 ratio. The Revised Atlanta Classification was used to diagnose AP.

**Results:**

Among 450 patients with IGB, 25 developed AP, yielding an incidence of 5.56%. The Orbera balloon was associated with a lower AP risk (HR 0.29, 95% CI: 0.09–0.96; *P* = 0.042). The median time to AP onset was 40 days. Higher preprocedural BMI and age > 30 years showed a trend toward reduced AP risk, though not statistically significant.

**Conclusions:**

AP following IGB insertion is uncommon but may be underreported, with substantial variability in onset time. The Orbera balloon demonstrated a protective effect, highlighting the role of balloon type in AP risk. These findings underscore the importance of balloon selection and the need for further prospective studies to confirm these results and optimize AP risk management in IGB patients.

**Supplementary Information:**

The online version contains supplementary material available at 10.1007/s11695-024-07647-x.

## Introduction

The prevalence of obesity (BMI ≥ 30) has increased substantially in recent decades, contributing to a wide range of comorbidities, including hypertension, type 2 diabetes, and obstructive sleep apnea [1, 2]. Even modest weight loss of 5–10% has been associated with significant improvements in these conditions [3–5]. Various treatment modalities are available to manage obesity, ranging from behavioral interventions and pharmacotherapy to surgical options [2, 6, 7]. One minimally invasive option is the intragastric balloon (IGB), designed to reduce gastric capacity and promote satiety [8]. IGBs are indicated for patients with a BMI ≥ 27 kg/m^2^ who are not candidates for bariatric surgery or who require a bridge to surgery due to extreme obesity (BMI ≥ 50 kg/m^2^) [9]. People who use an IGB can lose up to 15% of their total body weight, which is considerably more than the 1–5% typically achieved through lifestyle modifications alone. However, there are still concerns regarding the potential side effects associated with this method [10–12].

The most frequently reported side effects of IGB therapy include nausea and vomiting, which typically resolve within weeks after insertion [10, 11]. However, more serious complications, such as gastric ulceration, balloon rupture, and acute pancreatitis (AP), although rare, have also been reported. Since 2015, the FDA has documented approximately 30 cases of AP associated with IGBs [13]. A recent analysis of the FDA MAUDE database reported that severe complications, including AP, accounted for ≤ 1% of cases [14]. The mechanisms underlying IGB-associated AP remain incompletely elucidated but potentially involve mechanical pressure exerted by the balloon on the pancreas or duodenal compression, resulting in pancreatic obstruction and subsequent pancreatic injury [12]. Although AP is rare, it can lead to significant morbidity, and the current literature on its occurrence is limited to small case series, with the largest study involving just 10 patients [13].

The present study employed a retrospective cohort design to systematically investigate the incidence, predictors, and outcomes of AP after IGB insertion. By examining a large, multi-year dataset, this study provides a comprehensive framework for addressing existing gaps in the literature and offers evidence-based insights to inform clinical decision-making.

## Methods

### Study Design

This 7-year retrospective time-to-event study investigated the primary outcome: risk factors for developing AP after IGB insertion. A 1:3 AP to non-AP ratio was used to enhance statistical power, given the rarity of AP cases. Simple random sampling minimized sampling bias for the non-AP group. The incidence of AP was also calculated as a secondary outcome.

### Data Collection and Patient Selection

Data were collected from medical records, including patient demographics (age, sex, and BMI), balloon characteristics (type and volume), and lifestyle factors (alcohol consumption and smoking). Patients aged ≥ 18 years who underwent IGB insertion between January 2017 and 2024 were included in the study. AP diagnosis was confirmed using the Revised Atlanta Classification, which requires at least two of the following: characteristic epigastric pain, serum lipase and/or amylase levels > 3 times the upper limit of normal (ULN), and imaging evidence of AP via abdominal ultrasonography [15]. Patients in the non-AP group were required to have had IGB for a minimum of 6 months. Patients with a history of acute or chronic pancreatitis or gastrointestinal surgery prior to balloon insertion were excluded. Patients were followed from the time of IGB insertion until either the development of AP, removal of the balloon, or the conclusion of the study period. Abdominal ultrasonography was performed to exclude gallbladder stones or sludge.

### Statistical Analysis

Continuous variables were summarized as mean ± standard deviation (SD) or median with interquartile range (IQR). Categorical variables were reported as frequencies and percentages. The Wilcoxon rank-sum test was used for continuous data, whereas the chi-squared or Fisher’s exact test was used for categorical data.

Cox regression was employed to evaluate time-dependent risk factors for AP, as it accounts for varying follow-up times and provides hazard ratio (HR) estimates for potential predictors. The analysis included variables such as sex, age (> 30 years), pre-BMI (≥ 35), ethnicity, place of insertion (outside), alcohol use, GLP-1 medication use, smoking, type of balloon (Orbera), and fluid-filled balloons. The balloon volume variable was excluded due to 35% missing data, which could lead to overestimation.

To complement the Cox regression analysis, an exploratory logistic regression was performed on the same covariates. Logistic regression was used to evaluate the overall association between potential predictors and IGB-AP, regardless of time dependency, providing odds ratio (OR) estimates. This approach allowed for cross-validation of findings and ensured the robustness of results. The results were expressed as HR and OR with 95% confidence intervals (CI). Statistical significance was defined as a two-sided *P*-value of < 0.05. We conducted a sensitivity analysis, including and excluding the balloon volume variable due to its high proportion of missing data.

All statistical analyses were performed using the statistical packages SPSS version 29.0 (Armonk, NY, IBM Corp) and Epi Info 2000 (Centers for Disease Control and Prevention, Atlanta, GA, USA).

### Ethical Approval

All methods followed the Declaration of Helsinki and the hospital guidelines and regulations. The ethical committee waived the need for consent as only anonymous data without patient identifiers were provided to the research team. The study was approved by the Ethics and Research Committee of the Medical Research Center of Hamad Medical Corporation, Doha, Qatar (approval number MRC-01–24–270).

## Results

### Baseline Characteristics and Incidence of AP

Over 7 years, this study identified 25 cases of AP among 450 patients with IGB followed up at our center, yielding an incidence rate of approximately 5.56%. The baseline characteristics of 25 patients with AP and 75 patients without AP are presented in Table 1. Regarding the age of the cohort, 64% of the AP cohort was ≤ 30 years old compared with 41% of the non-AP group (*P* = 0.05). The majority of the cohort was female (66%), which was similar in both groups. Regarding BMI at IGB insertion, the predominant measurement was BMI 35–40 (71.7%), which was equally distributed in both groups. Regarding IGB characteristics, the Orbera type was predominant and employed in 85.7% of the cohort, with a higher frequency in the non-AP group than in the AP group (89% vs. 74%, *P* = 0.06). Box plots depicting the distribution of age, BMI, and balloon volume across sex and AP and non-AP groups are shown in supplementary Fig. S1–S3.

**Table 1:** Demographic and clinical characteristics of patients with and without acute pancreatitis following intragastric balloon insertion

Variable	Overall (*n* = 100)	IGB with AP (*n* = 25)	IGB without AP (*n* = 75)	*P*-value
Age (years): *n* (%)				0.049
≤ 30 years	47 (47)	16 (64)	31(41.3)	
> 30 years	53 (53)	9 (36)	44 (58.7)	
Sex: female *n* (%)	66 (66)	16 (64)	50 (66.7)	0.807
Ethnicity: Arab, yes *n* (%)	76 (76)	22 (88)	54 (72)	0.105
BMI at IGB insertion time (kg/m^2^) * *n* (%)				0.460
< 35	10 (10.9)	4 (17.4)	6 (8.7)	
35–40	66 (71.7)	16 (69.6)	50 (72.5)	
> 40	16 (17.4)	3 (13)	13 (18.8)	
Smoking: yes *n* (%)	13 (13)	3 (12)	10 (13.3)	0.864
Alcohol: yes *n* (%)	4 (4)	1 (4)	3 (4.0)	0.990
GLP-1 medication: yes *n* (%)	9 (9)	1 (4)	8 (10.7)	0.313
Type of IGB*: *n* (%)				0.064
Orbera	84 (85.7)	17 (73.9)	67 (89.3)	
Non-Orbera	14 (14.3)	6 (26.1)	8 (10.7)	
Place of IGB insertion: *n* (%)				0.812
Our center *n* (%)	38 (38)	10 (40)	28 (37.3)	
Another centers *n* (%)	62 (62)	15 (60)	47 (62.7)	
Fluid-filled: yes *n* (%)	90 (90)	22 (88)	72 (96)	0.941
Volume of balloon (ml): *n* (%)				0.424
< 500	11 (17.5)	5 (23.8)	6 (14.3)	
500–600	40 (63.5)	11 (52.4)	29 (63.5)	
> 600	12 (19)	5 (23.8)	7 (19)	

^*^Note: Percentages and totals may not sum to 100% or the full sample size due to missing data

### Further Characteristics of the AP Group

Pancreatitis severity was classified as mild in all cases. Regarding laboratory findings during AP, the median lipase level was 344 U/L (IQR: 206–1217 U/L), and the median amylase level was 128 U/L (IQR: 69–342 U/L). Abdominal ultrasonography was performed in 22 patients (88%) to exclude the presence (GB) stones. One case revealed GB stones, and two cases demonstrated GB sludge; nevertheless, cholecystectomy was not indicated.

The IGB type was Orbera in 17 (74%) patients. The non-Orbera group included six patients: three with Spatz adjustable IGB, one with Medsil IGB, one with END BALL IGB, and one with air-filled Ellipse IGB. The type of IGB was unknown in two cases due to missing data. A single complication occurred in this group, and balloon rupture necessitated urgent IGB removal. Follow-up of this population for up to 1 year after IGB removal did not reveal any recurrence of AP, even in the case of GB stones (Table 2).

**Table 2:** Detailed characteristics of the acute pancreatitis group following intragastric balloon insertion (*n* = 25)

Variable	Results
Age (mean ± SD)	24.5 ± 10
AP onset, days (median, IQR)	40 (9.5–195)
BMI at IGB insertion time (kg/m^2^) (mean ± SD)	34 ± 6.03
BMI at pancreatitis time (kg/m^2^) (mean ± SD)	30 ± 6.30
Pancreatitis severity** (mild), yes *n* (%)	25 (100)
Abdominal pain: yes *n* (%)	25 (100)
Fever: yes *n* (%)	0 (0)
Lipase (U/L) (median, IQR)	344 (206–1217)
Amylase (U/L) (median, IQR)	128 (69–342)
CRP (mg/L) (median, IQR)	6.8 (2.3–19.5)
Hypertriglyceridemia: yes *n* (%)	0 (0)
US abdomen: yes *n* (%)	22 (88)
GB stones/sludge by US: yes *n* (%)	3 (12)
CT abdomen: yes *n* (%)	4 (16)
Type of IGB*: *n* (%)	
Orbera	17 (73.9)
Non-Orbera	6 (26.1)
Volume of balloon (ml) (mean ± SD)	584 ± 76.2
Balloon removed: yes *n* (%)	21 (84)
In-hospital complications: yes *n* (%)	1 (4)
LOS (median, IQR)	2 (2–4)
Recurrent AP up to 1 year: yes *n* (%)	0 (0)

^*^Note: Percentages and totals may not sum to 100% or the full sample size due to missing data

^**^Severity of pancreatitis was classified based on the Revised Atlanta Classification (15)

### Time to Onset of Acute Pancreatitis: Kaplan–Meier Analysis

**Fig. 1:**
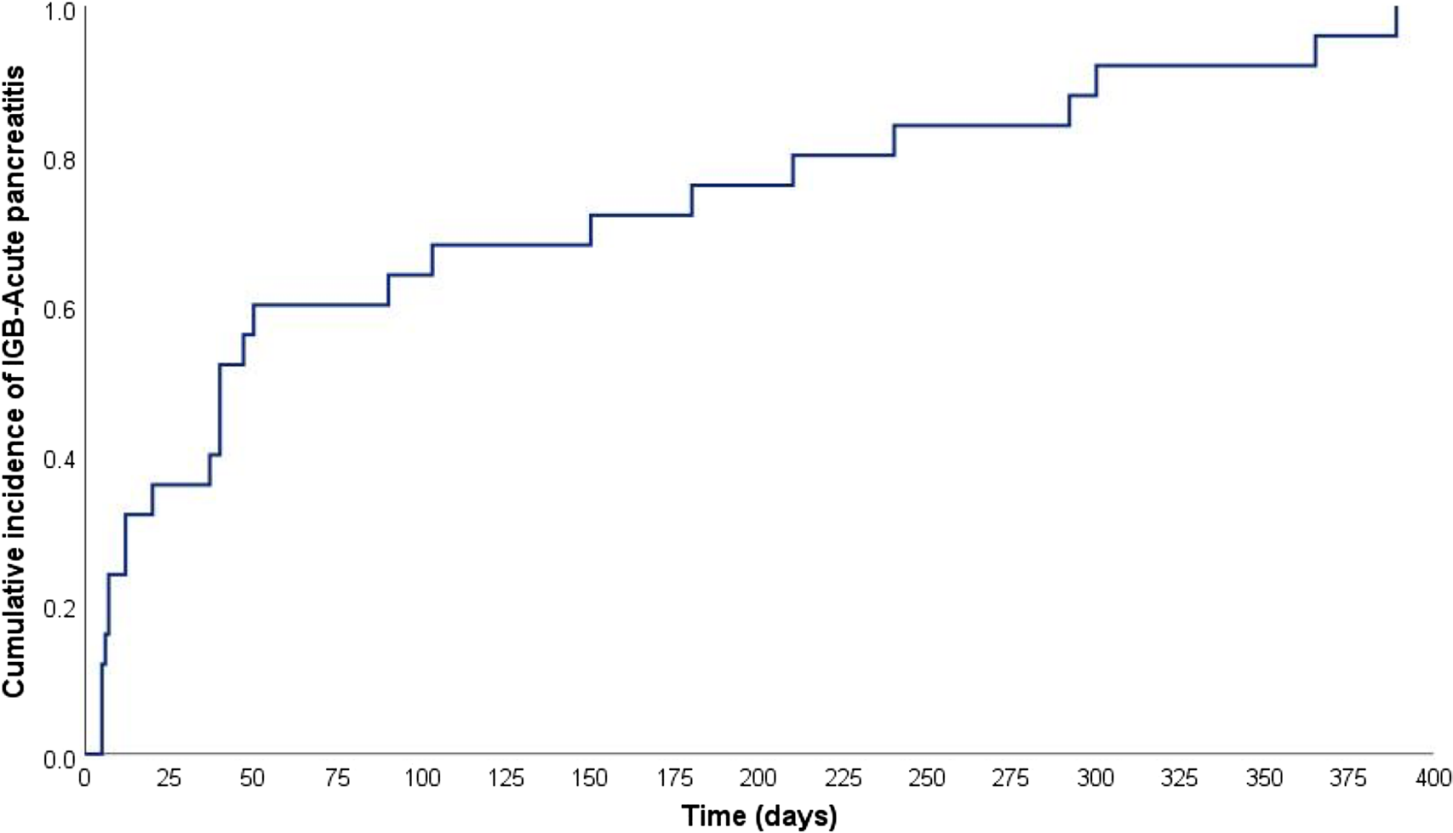
Kaplan–Meier curve for time to IGB-associated AP onset. The median time to AP onset was 40 days (95% CI: 27.8–52.2 days), with 25% of patients developing AP within 12 days and 75% by 180 days

**Fig. 2:**
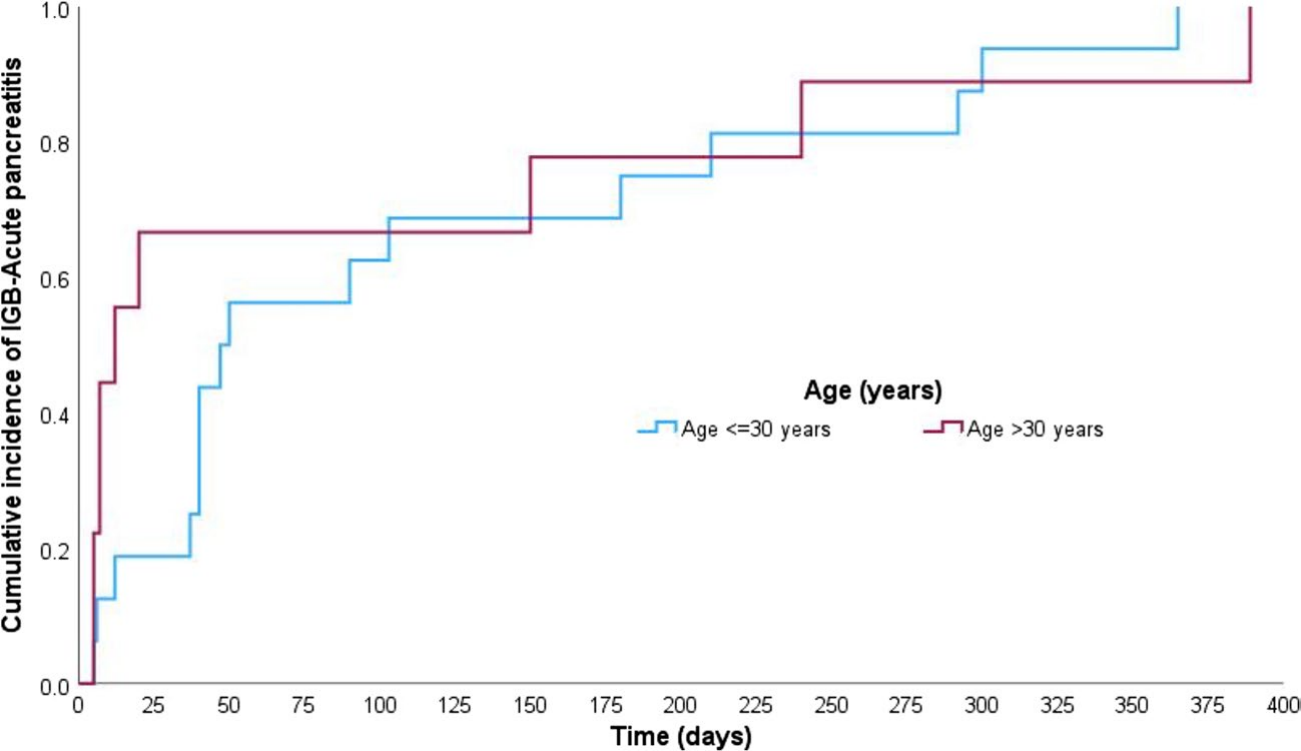
Kaplan–Meier curve comparing AP onset by age group. Patients over 30 years had a shorter median time to AP onset (12 days) compared to those 30 or younger (47 days), indicating age as a potential factor influencing AP onset timing

**Fig. 3:**
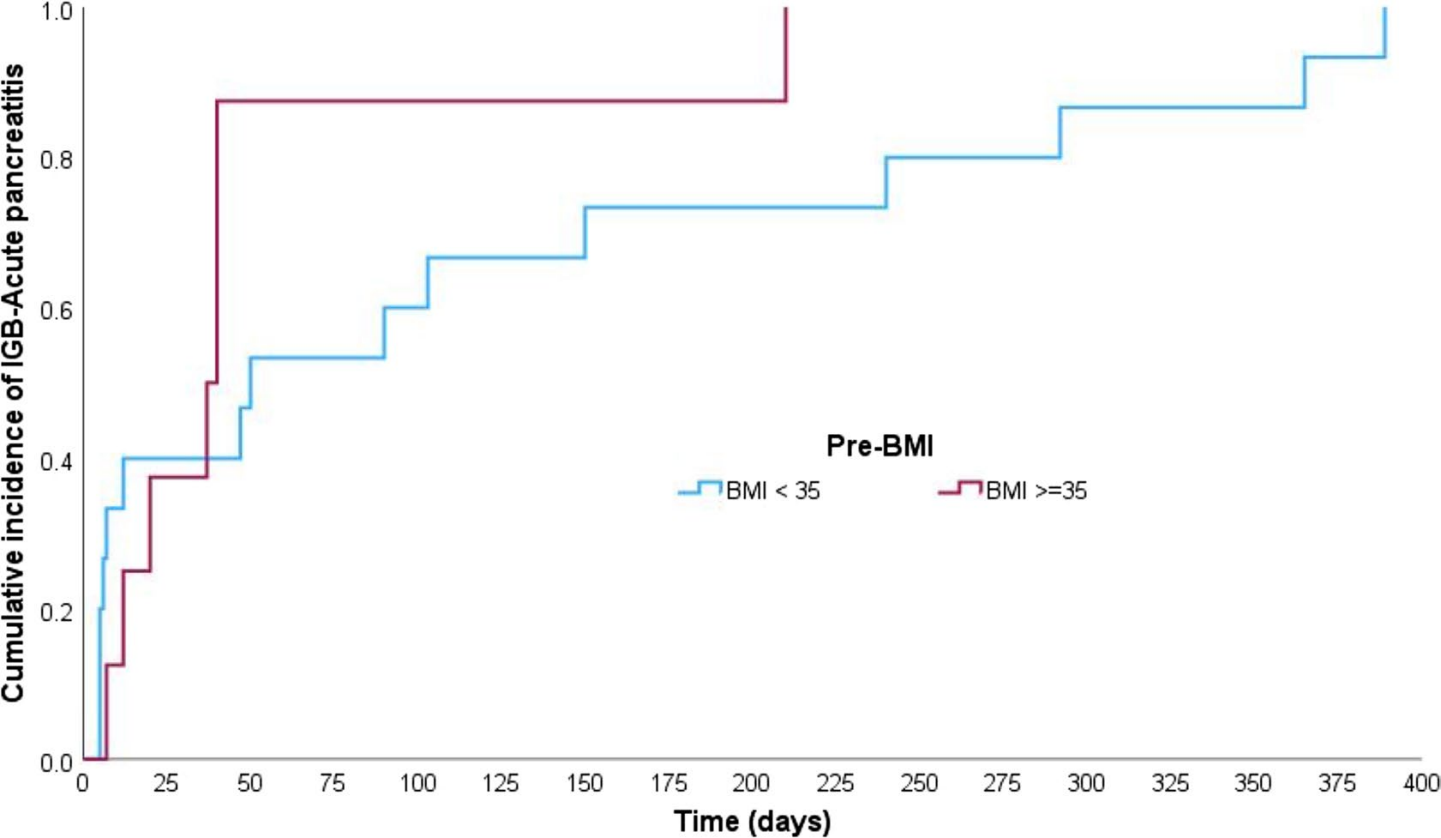
Kaplan–Meier curve comparing AP onset by BMI category. Patients over BMI ≥ 35 had a shorter median time to AP onset (37 days) than those with BMI < 35 (50 days), suggesting that higher BMI may be associated with earlier AP onset

**Fig. 4:**
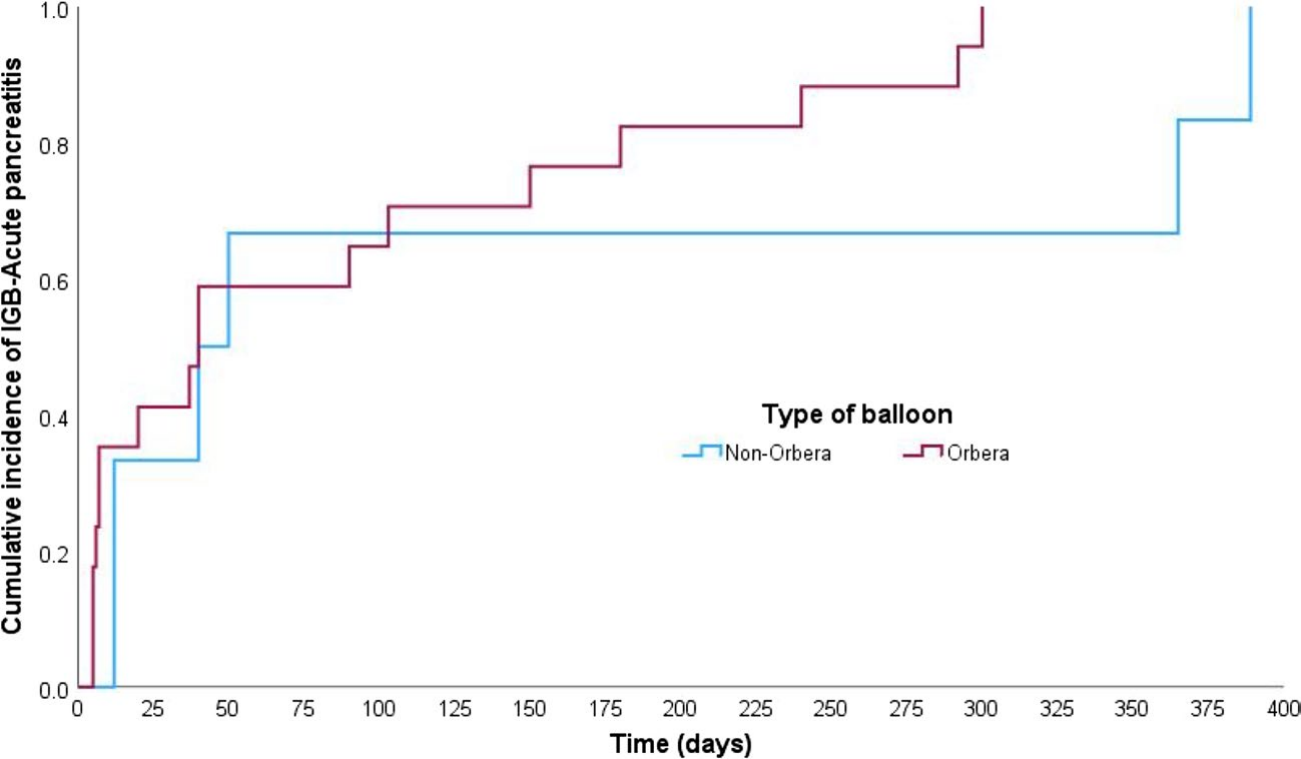
Kaplan–Meier curve comparing AP onset by balloon type (Orbera vs. non-Orbera). Both groups had a similar median time of 40 days to AP onset, indicating that the type of balloon did not markedly impact AP onset timing

The Kaplan–Meier analysis (Figs. 1, 2, 3, and 4) demonstrated that the median time to IGB-AP was 40 days (95% CI: 27.8–52.2 days), with 25% of patients developing AP within 12 days and 75% by 180 days. Variations were observed across demographic categories; patients aged > 30 years (Fig. 2) exhibited a shorter median time to AP onset (12 days) than those aged ≤ 30 years (47 days). Similarly, individuals with a BMI ≥ 35 (Fig. 3) developed AP earlier (median, 37 days) than those with a BMI < 35 (median, 50 days). Notably, the balloon type (Orbera vs. non-Orbera; Fig. 4) did not demonstrate a substantial difference, with both groups presenting a similar median time of 40 days to AP onset.

### Risk Factors for AP: Cox and Logistic Regression Analysis

In both Cox regression and logistic regression analyses (Tables 3 and 4), specific factors were associated with a reduced risk of AP following IGB insertion. The Orbera balloon demonstrated a protective effect (HR 0.29, 95% CI: 0.09–0.96; *P* = 0.042; OR 0.13, 95% CI: 0.03–0.66; *P* = 0.013) compared to other balloon types, after adjusting for potential predictors and covariates. Additionally, IGB insertion outside the primary setting was linked to a lower hazard of AP in Cox regression (HR 0.34, 95% CI: 0.12–0.97; *P* = 0.043). Sensitivity analysis, including and excluding the balloon volume variable, confirmed consistent results (Supplementary Tables 1–2). Although not statistically significant (*P* > 0.05), a higher preprocedural BMI (≥ 35) and age > 30 years indicated a trend towards reduced AP risk in both analyses, suggesting a potential protective role.

**Table 3:** Univariate and multivariate Cox regression analysis of predictors for acute pancreatitis following intragastric balloon insertion

		**Univariate analysis**				**Multivariate analysis**		
**Predictors**	Unadjusted hazard ratio (HR)	95% CI for unadjusted HR		*P*-value	Adjusted hazard ratio (HR)	95% CI for adjusted HR		*P*-value
		Lower	Upper			Lower	Upper	
Sex (female)	0.86	0.38	1.96	0.726	0.45	0.14	1.43	0.175
Age (> 30 years)	0.54	0.24	1.24	0.146	0.51	0.19	1.35	0.173
Pre-BMI (≥ 35)	0.60	0.26	1.42	0.246	0.43	0.16	1.15	0.093
Ethnicity (others)	0.41	0.12	1.37	0.148	0.44	0.10	1.94	0.279
Place of insertion (outside)	0.71	0.31	1.61	0.413	0.34	0.12	0.97	0.043
Alcohol (yes)	0.85	0.11	6.32	0.873	3.57	0.17	76.44	0.416
GLP—1 medication (yes)	0.44	0.06	3.23	0.415	0.36	0.04	3.00	0.345
Smoking (yes)	0.89	0.27	2.97	0.846	0.28	0.03	2.34	0.240
Type of balloon (Orbera)	0.48	0.19	1.22	0.123	0.29	0.09	0.96	0.042
Fluid-filled (yes)	0.77	0.10	5.76	0.798	2.18	0.22	21.70	0.508

**Table 4:** Univariate and multivariate logistic regression analysis of predictors for acute pancreatitis following intragastric balloon insertion

		**Univariate analysis**				**Multivariate analysis**		
**Predictors**	Unadjusted odds ratio (OR)	95% CI for unadjusted OR		*P*-value	Adjusted odds ratio (OR)	95% CI for adjusted OR		*P*-value
		Lower	Upper			Lower	Upper	
Sex (female)	0.89	0.35	2.29	0.807	0.48	0.12	1.92	0.296
Age (> 30 years)	0.40	0.16	1.01	0.053	0.36	0.10	1.22	0.099
Pre-BMI (≥ 35)	0.52	0.20	1.38	0.188	0.29	0.09	1.02	0.053
Ethnicity (others)	0.35	0.10	1.30	0.116	0.45	0.08	2.70	0.381
Place of insertion (outside)	0.89	0.35	2.26	0.812	0.30	0.09	1.07	0.063
Alcohol (yes)	0.99	0.10	10.07	0.990	8.52	0.24	306.24	0.241
GLP—1 medication (yes)	0.35	0.04	2.94	0.333	0.27	0.02	3.05	0.287
Smoking (yes)	0.89	0.22	3.52	0.864	0.17	0.01	2.37	0.190
Type of balloon (Orbera)	0.34	0.10	1.11	0.073	0.13	0.03	0.66	0.013
Fluid-filled (yes)	0.92	0.09	9.26	0.941	4.95	0.27	89.69	0.279

## Discussion

Over 7 years, this study identified 25 cases of IGB-related AP, primarily in young females (mean age 24.5 years, BMI 34). This demographic profile aligns with previous IGB-AP reports [13, 16–19], as young women are more likely to pursue weight management interventions due to societal expectations, self-image concerns, and health considerations [20, 21]. The incidence of AP in our study was 5.56%, which is significantly higher than that reported in a recent meta-analysis (0.006%) [22]. It is important to note that our study was not designed to calculate AP incidence as a primary outcome. This discrepancy with previous studies may be due to underreporting, as AP symptoms often mimic routine post-IGB discomfort, leading to delays in seeking care. As the primary center for post-IGB complications in the country, we may have captured more cases, potentially overestimating the incidence. In contrast, lower AP rates in earlier studies may reflect smaller sample sizes, shorter follow-up periods, or a reliance on voluntary reporting systems. Additionally, ethnic or genetic factors may have contributed to the observed differences.

Cox and logistic regression analyses identified balloon type as a factor associated with AP, with the Orbera balloon showing a lower hazard rate compared to other types. While prior case reports and series indicated more Orbera cases [13, 16], our analysis revealed the opposite, emphasizing the importance of rigorous statistical methods in identifying true risk factors. Furthermore, IGB insertion outside the primary setting was linked to a lower AP risk, potentially due to differences in procedural techniques, follow-up, or patient characteristics. However, unmeasured confounders related to insertion location may also play a role. Although age and higher BMI appeared to have a protective effect against AP, the findings did not reach statistical significance. Age-related pancreatic changes, such as steatosis, fibrosis, and enzyme reduction, may lower inflammation [23]. Additionally, visceral adipose tissue in individuals with higher BMI might provide mechanical protection to the pancreas [24]. While plausible, these hypotheses remain speculative and require further investigation.

The duration from IGB insertion to AP exhibited substantial variability among cases, with a median of 40 days post-insertion. Similar variability has been observed in previous case reports and series [13, 16]. While no definitive explanation exists for this variability, our findings emphasize the necessity of vigilant monitoring of patients following IGB insertion, not only in the initial weeks, as AP can occur at any point throughout the post-insertion period. IGB removal was performed in most cases, aligning with reported IGB-AP management [13, 16]. While no consensus exists for mandatory removal in mild AP, Brazilian and Spanish consensus (based on 20,000–40,000 cases) suggest it may not be necessary, leaving the decision to clinical judgment on a case-by-case basis [25, 26].

To address potential confounders, we investigated other AP causes. Abdominal ultrasonography identified one case of gallbladder stones and two with sludge. However, no recurrent AP episodes occurred over 1 year despite gallbladders remaining in situ. This observation is significant, as patients with gallbladders in situ have been shown to have up to a 45% recurrence rate within 6 months after initial biliary pancreatitis [27, 28], suggesting that biliary etiology is unlikely in our cases. Additionally, only one patient reported occasional alcohol use, with no AP recurrence over 1 year, reducing alcohol as a likely factor. No cases of hypertriglyceridemia were identified.

This study has several strengths. To the best of our knowledge, this is the largest study on IGB-related AP to date. Time-to-event analysis and Cox regression provided key insights into the timing and risk factors for AP, while extended follow-up (up to 1 year) allowed the detection of recurrent episodes and comprehensive patient outcomes. This study offers a robust evaluation of AP risk by examining various confounding factors, including IGB type. Despite its strengths, this study has several limitations. The sample size, although the largest for IGB-related AP, remained relatively small. The predominant use of the Orbera balloon introduces selection bias, limiting its generalizability to other balloon types. As a retrospective study, it is subject to biases (information, selection, and observer bias) that may affect data accuracy. Additionally, missing variables, such as balloon volume, restricted our analysis and may have influenced our findings.

## Conclusion

Acute pancreatitis following intragastric balloon insertion is an uncommon but noteworthy complication with generally favorable outcomes, although it may be underreported owing to overlapping symptoms with typical postprocedural effects. The time to onset of acute pancreatitis varied significantly, underscoring the need for close observation throughout balloon placement. Cox regression analysis indicated a potential protective effect of the Orbera balloon compared with other balloon types. Clinically, these findings may guide balloon-type selection and inform future monitoring strategies. Future research, particularly larger prospective studies, could improve our understanding of acute pancreatitis risk in intragastric balloon patients, ultimately supporting safer patient selection and more refined management strategies.

## Supplementary Information

Below is the link to the electronic supplementary material.

Supplementary file1 (DOCX 186 KB)

## Data Availability

No datasets were generated or analysed during the current study.

## Abbreviations

AP: Acute pancreatitis
BMI: Body mass index
CI: Confidence interval
FDA: Food and Drug Administration
GB: Gallbladder
GLP-1: Glucagon-like peptide-1
HR: Hazard ratio
IGB: Intragastric balloon
IQR: Interquartile range
LOS: Length of stay
MAUDE: Manufacturer and User Facility Device Experience
SD: Standard deviation
SPSS: Statistical Package for the Social Sciences
ULN: Upper limit of normal
