# Supplementary material for: Risk Factors for Acute Pancreatitis Following Intragastric Balloon Insertion: A 7-Year Retrospective Cohort Study
Source: Obes Surg. 2025 Jan 14;35(2):496–504. doi: 10.1007/s11695-024-07647-x (PMC11836173; doi:10.1007/s11695-024-07647-x)
Supplement: Supplementary file 1 — Supplementary file1 (DOCX 186 KB) [file 11695_2024_7647_MOESM1_ESM.docx]

**Supplementary Figures – Sex, Age, BMI, and Balloon Volume in Acute Pancreatitis Post-Intragastric Balloon Insertion**


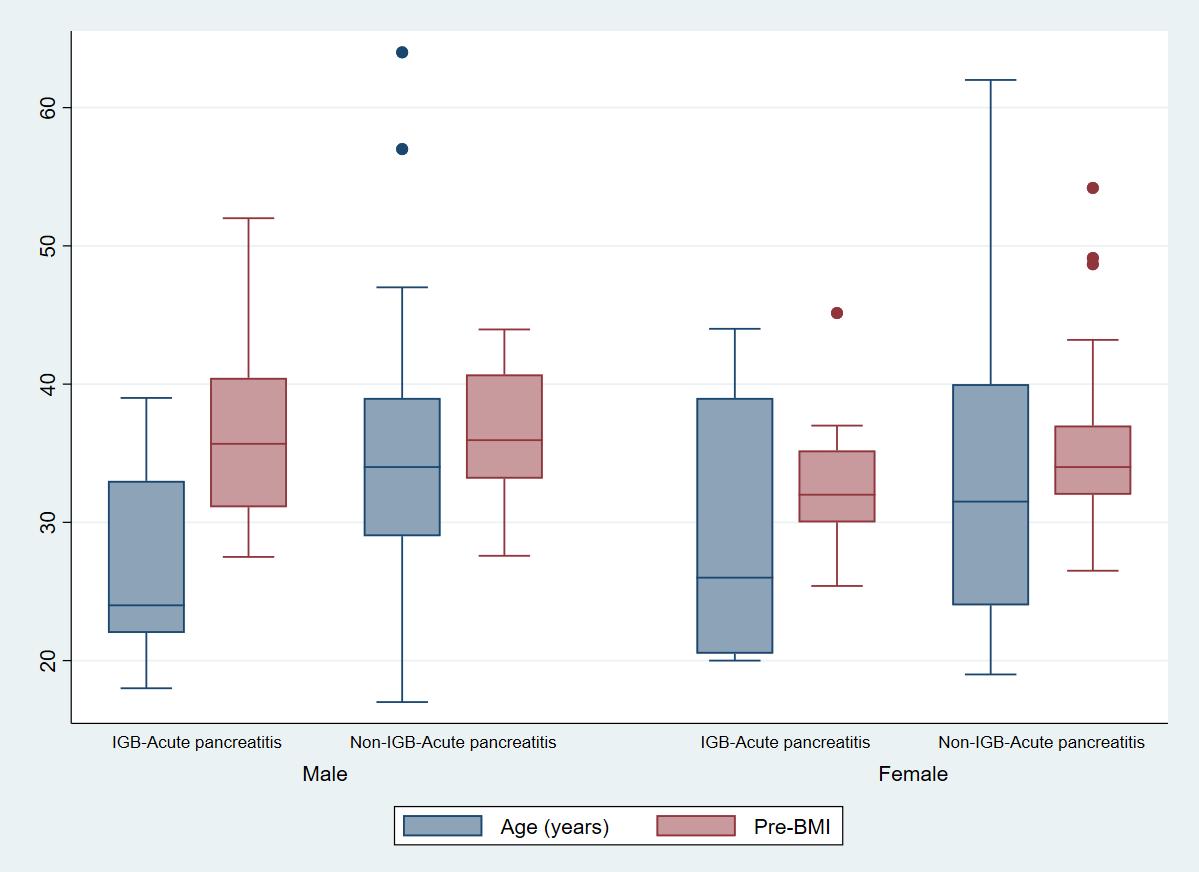


*Supplementary Figure S1. Sex Distribution, Age, and Pre-IGB BMI Among Patients with AP Post-IGB*


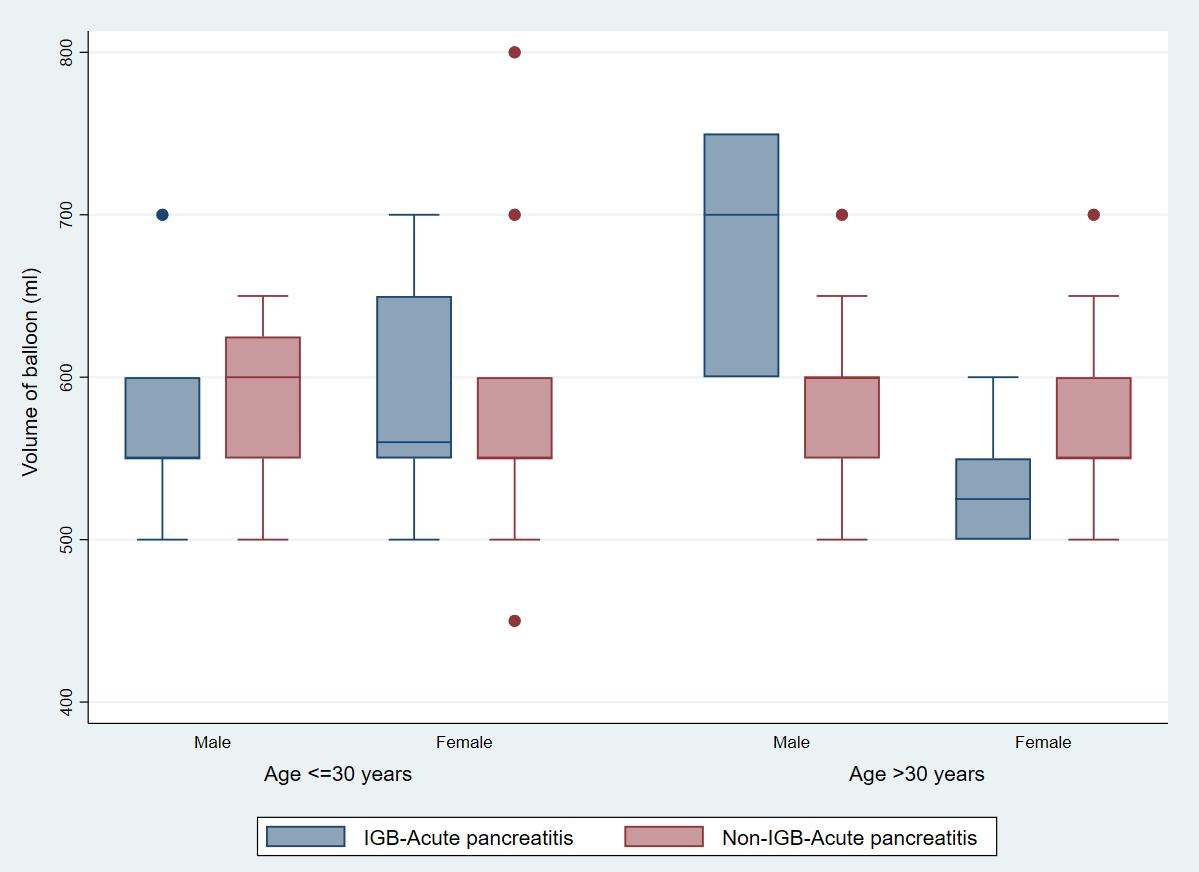


*Supplementary Figure S2. Balloon Volume by Sex, Age Group, and AP Status in Patients Post-IGB*


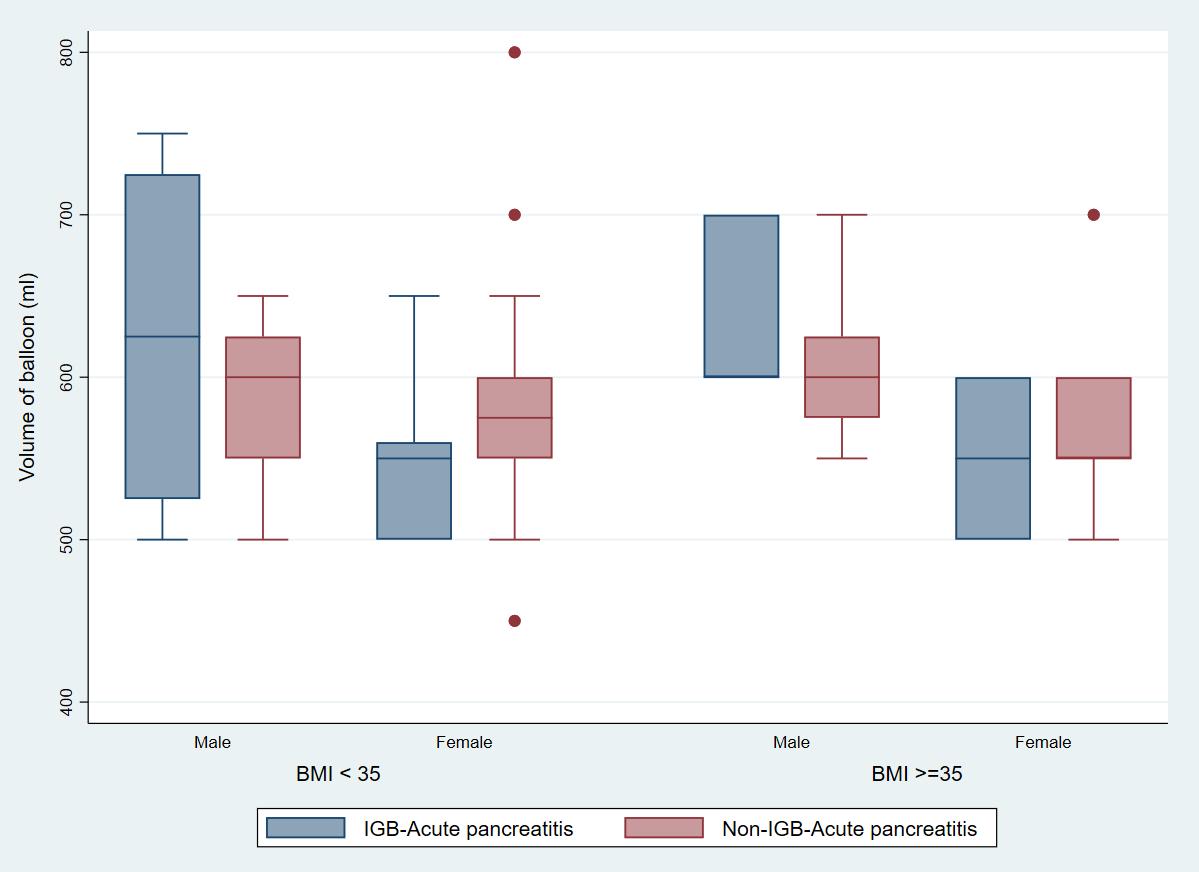


*Supplementary Figure S3. Balloon Volume by Sex, BMI Category, and AP Status in Patients Post-IGB*

|  |  | ***Univariate Analysis*** | |  |  | |  | ***Multivariate Analysis*** | |  |
| --- | --- | --- | --- | --- | --- | --- | --- | --- | --- | --- |
| ***Predictors*** | *Unadjusted*  *Hazard*  *ratio (HR)* | *95% CI for*  *Unadjusted HR* | | *P-value* | |  | *Adjusted Hazard ratio (HR)* | *95% CI for*  *Adjusted HR* | | *P-value* |
|  |  | *Lower* | *Upper* |  |  |  |  | *Lower* | *Upper* |  |
| *Gender (female)* | *0.86* | *0.38* | *1.96* | *0.726* | |  | *0.28* | *0.07* | *1.07* | *0.063* |
| *Age (>30 years)* | *0.54* | *0.24* | *1.24* | *0.146* | |  | *0.58* | *0.20* | *1.68* | *0.318* |
| *Pre-BMI (≥35)* | *0.60* | *0.26* | *1.42* | *0.246* | |  | *0.48* | *0.14* | *1.64* | *0.240* |
| *Volume of balloon (>550 ml)* | *0.89* | *0.38* | *2.09* | *0.780* | |  | *0.47* | *0.16* | *1.38* | *0.169* |
| *Ethnicity (Others)* | *0.41* | *0.12* | *1.37* | *0.148* | |  | *0.78* | *0.16* | *3.77* | *0.754* |
| *Place of insertion (outside)* | *0.71* | *0.31* | *1.61* | *0.413* | |  | *0.20* | *0.05* | *0.79* | ***0.022*** |
| *Alcohol (yes)* | *0.85* | *0.11* | *6.32* | *0.873* | |  | *1.67* | *0.07* | *41.59* | *0.756* |
| *GLP - 1 medication (yes)* | *0.44* | *0.06* | *3.23* | *0.415* | |  | *0.24* | *0.03* | *2.15* | *0.204* |
| *Smoking (yes)* | *0.89* | *0.27* | *2.97* | *0.846* | |  | *0.22* | *0.02* | *2.53* | *0.223* |
| *Type of balloon (Orbera)* | *0.48* | *0.19* | *1.22* | *0.123* | |  | *0.12* | *0.03* | *0.56* | ***0.007*** |
| *Fluid filled (yes)* | *0.77* | *0.10* | *5.76* | *0.798* | |  | *3.35* | *0.30* | *37.42* | *0.326* |

***Supplementary Table 1 Univariate and Multivariate Cox Regression Analysis (including Volume of Balloon)***

***Supplementary Table 2 Univariate and Multivariate Logistic Regression Analysis (including Volume of balloon)***

|  |  | ***Univariate Analysis*** | |  |  | ***Multivariate Analysis*** | | |  |
| --- | --- | --- | --- | --- | --- | --- | --- | --- | --- |
| ***Predictors*** | *Unadjusted Odds ratio (OR)* | *95% CI for*  *Unadjusted OR* | | *P-value* | *Adjusted Odds ratio (OR)* | *95% CI for*  *Adjusted OR* | | *P-value* | |
|  |  | *Lower* | *Upper* |  |  | *Lower* | *Upper* |  |  |
| *Gender (female)* | *0.89* | *0.35* | *2.29* | *0.807* | *0.16* | *0.03* | *1.05* | *0.057* | |
| *Age (>30 years)* | *0.40* | *0.16* | *1.01* | *0.053* | *0.58* | *0.12* | *2.76* | *0.491* | |
| *Pre-BMI (≥35)* | *0.52* | *0.20* | *1.38* | *0.188* | *0.18* | *0.03* | *1.12* | *0.066* | |
| *Volume of balloon (>550 ml)* | *0.83* | *0.29* | *2.34* | *0.722* | *0.38* | *0.08* | *1.80* | *0.221* | |
| *Ethnicity (Others)* | *0.35* | *0.10* | *1.30* | *0.116* | *0.92* | *0.12* | *7.26* | *0.940* | |
| *Place of insertion (outside)* | *0.89* | *0.35* | *2.26* | *0.812* | *0.12* | *0.02* | *0.78* | ***0.026*** | |
| *Alcohol (yes)* | *0.99* | *0.10* | *10.07* | *0.990* | *3.74* | *0.04* | *356.48* | *0.570* | |
| *GLP - 1 medication (yes)* | *0.35* | *0.04* | *2.94* | *0.333* | *0.17* | *0.01* | *2.29* | *0.181* | |
| *Smoking (yes)* | *0.89* | *0.22* | *3.52* | *0.864* | *0.04* | *0.00* | *2.92* | *0.138* | |
| *Type of balloon (Orbera)* | *0.34* | *0.10* | *1.11* | *0.073* | *0.01* | *0.00* | *0.19* | ***0.003*** | |
| *Fluid filled (yes)* | *0.92* | *0.09* | *9.26* | *0.941* | *57.63* | *0.78* | *4284.16* | *0.065* | |
